# Clinical metagenomics—challenges and future prospects

**DOI:** 10.3389/fmicb.2023.1186424

**Published:** 2023-06-28

**Authors:** Maliha Batool, Jessica Galloway-Peña

**Affiliations:** Department of Veterinary Pathobiology, College of Veterinary Medicine and Biomedical Sciences, Texas A&M University, College Station, TX, United States

**Keywords:** metagenomics, sepsis, diagnostics, meningitis, next-generation sequencing

## Abstract

Infections lacking precise diagnosis are often caused by a rare or uncharacterized pathogen, a combination of pathogens, or a known pathogen carrying undocumented or newly acquired genes. Despite medical advances in infectious disease diagnostics, many patients still experience mortality or long-term consequences due to undiagnosed or misdiagnosed infections. Thus, there is a need for an exhaustive and universal diagnostic strategy to reduce the fraction of undocumented infections. Compared to conventional diagnostics, metagenomic next-generation sequencing (mNGS) is a promising, culture-independent sequencing technology that is sensitive to detecting rare, novel, and unexpected pathogens with no preconception. Despite the fact that several studies and case reports have identified the effectiveness of mNGS in improving clinical diagnosis, there are obvious shortcomings in terms of sensitivity, specificity, costs, standardization of bioinformatic pipelines, and interpretation of findings that limit the integration of mNGS into clinical practice. Therefore, physicians must understand the potential benefits and drawbacks of mNGS when applying it to clinical practice. In this review, we will examine the current accomplishments, efficacy, and restrictions of mNGS in relation to conventional diagnostic methods. Furthermore, we will suggest potential approaches to enhance mNGS to its maximum capacity as a clinical diagnostic tool for identifying severe infections.

## 1. Introduction

### 1.1. Overview of the limitations of routine diagnostics for pathogen detection

Conventionally, the clinical detection of pathogens is based on the isolation and cultivation of organisms (Fournier et al., 2014). Once cultivated, these organisms are typically characterized using biochemical tests, mass spectrometry, nuclear magnetic resonance (NMR) spectrometry, nucleic acid amplification, or immunological testing (Carroll et al., 2019). Culture-dependent methods are considered the “gold standard” for diagnosis of infectious diseases in clinics but it may take several days to weeks to cultivate slow-growing organisms. Also, prior exposure to antibiotics can impair the sensitivity of culturing, thus missing cases of treatable diseases (Govender et al., 2021).

Polymerase chain reaction (PCR) is a widely used molecular diagnostic method in clinical laboratories that can rapidly detect the presence or absence of DNA and RNA from a clinical specimen without the need for microbial cultivation (Carroll et al., 2019). PCR-based tests have been further developed into real-time PCR, allowing the amplification, quantification of expression, and thus identification of specific pathogen genetic content with high sensitivity and specificity. However, PCR-based methodologies typically detect the presence or absence of a single gene at a time, offering low sensitivity, and potentially providing false negatives in cases containing low gene copy numbers (Huanyu Wang, 2021). To enhance the diagnostic capacity of PCR, multiplex PCR was developed to allow the simultaneous detection of multiple targets in a single PCR reaction (Huanyu Wang, 2021), although, it requires prior knowledge about pathogens of interest in order to identify them (Gu et al., 2021). Broad-range PCR is another effective method for hypothesis-independent detection of bacterial or fungal species, but has limitations. It has lower sensitivity than species-specific PCRs, cannot detect viral or parasitic infections, is only suitable for sterile bodily fluids and tissues, and can be more expensive than traditional methods (Rampini et al., 2011; Tkadlec et al., 2019; Aggarwal et al., 2020). Different PCR tests have varying diagnostic accuracy. In-house PCR tests are cheaper but require more time and training, while commercial PCR tests are automated and faster with higher sensitivity (Venter et al., 2019).

Alternatively, although antigen-detection is inexpensive and can be used in point-of-care setting due to promptness of the assay, diagnosis based on immunological tests are inherently less sensitive and may not provide accurate information (Govender et al., 2021). Furthermore, since it may take 1–2 weeks for antibodies to develop, antibody testing is not recommended for the diagnosis of acute disease (Govender et al., 2021).

In recent years, matrix-assisted laser desorption/ionization time-of-flight mass spectrometry (MALDI-TOF MS) has become a tool of choice for bacterial and fungal identification in clinical laboratories (Dingle and Butler-Wu, 2013). Although identification can be provided in minutes, MALDI-TOF requires bacterial cultivation prior to analysis. Furthermore, it is not a quantitative approach and presents low specificity (Roux-Dalvai et al., 2019).

Peptide nucleic acid fluorescent *in situ* hybridization (PNA-FISH) is a recently introduced rapid and reliable method for the detection of bacteria and fungi responsible for blood stream infections. It provides more timely results compared to traditional culturing-based methods (Calderaro et al., 2014). PNA-FISH is validated by the U.S. Food and Drug Administration (FDA) for diagnosis of blood samples, however, it is not available to use at the tissue level (Weaver et al., 2019). A summary of the current technologies used in routine diagnostics in the clinical setting is provided in Table 1.

**Table 1:** Routine diagnostic assays in a clinical setting for the detection of pathogens.

Technology	Manufacturer	Trade name	Detection time	Organisms	Limitations
**Respiratory infections**					
Multiplex PCR	Biofire Diagnostics, GenMark Diagnostics, Luminex, Roche Diagnostics	FilmArray, ePlex, VERIGENE, Lyra, Cobas Liat PCR system, NxTAG	15 min–2 h	Bacteria	
				*Streptococcus* spp*., Haemophilus influenzae, Neisseria meningitidis, Enterobacter* spp.*, Klebsiella* spp.*, Pseudomonas aeruginosa, Proteus* spp., *Serratia marcescens*	▪ Requires a separate primer set for each target gene
				Viruses	▪ Low application efficiency
				Adenovirus, coronavirus, human metapneumovirus, human rhinovirus/enterovirus, influenza A, influenza B, parainfluenza virus, respiratory syncytial virus, influenza A/B	
Nuclear magnetic resonance (NMR)	T2 biosystems	T2SARS-CoV-2	<2 h	SARS-CoV-2	▪ Magnetic field drift that may be detrimental to NMR spectra
**Bloodstream infections**					
Multiplex PCR	Biofire Diagnostics, GenMark Diagnostics	FilmArray BCID, ePlex BCID	1–1.5 h	Gram-negative bacteria	
				*Acinetobacter baumannii, Bacteroides fragilis, Citrobacter, Escherichia coli, Enterobacter* spp.*, Enterococcus spp., Fusobacterium* spp*., Haemophilus influenzae, Klebsiella* spp.*, Neisseria meningitidis, Pseudomonas aeruginosa, Proteus* spp.*, Serratia marcescens, Salmonella* spp*., Stenotrophomonas maltophilia*	▪ Requires background knowledge of suspected pathogen
				Gram-positive bacteria	
				*Bacillus* spp.*, Corynebacterium* spp.*, Cutibacterium acnes, Enterococcus spp., Listeria monocytogenes, Micrococcus* spp*., Staphylococcus* spp*., Streptococcus* spp.	▪ Unable to detect pathogens containing low copy number of genes in a clinical sample
				Fungal pathogens	
				*Candida* spp.*, Fusarium* spp*., Rhodotorula* spp.	
PNA-FISH	Accelerate Diagnostics, AdvanDx,	Accelerate Pheno, QuickFISH	20 min–1.5 h	Gram-negative bacteria	▪ Detects limited number of targets
				*Acinetobacter baumannii, Citrobacter* spp*., Escherichia coli, Enterobacter* spp*., Klebsiella* spp*., Pseudomonas aeruginosa, Proteus* spp*., Serratia marcescens*	
				Gram-positive bacteria	
				*Enterococcus* spp.*, Staphylococcus* spp.*, Streptococcus* spp.,	
				Fungal pathogens	
				*Candida* spp.	
MALDI-TOF	BioMerieux and Bruker	VITEK MS, MALDI Biotyper	30 min		▪ Has proprietary databases
					▪ Limited in the differentiation of closely related species
rRNA/PCR	Karius	Karius test		Detects greater than 1,000 pathogens	▪ Give false positives
Nuclear magnetic resonance	T2 Biosystems	T2Bacteria, T2Candida		Gram positive and gram-negative bacteria	▪ Low limit of detection
				*Escherichia coli, Pseudomonas aeruginosa, Klebsiella pneumoniae, Staphylococcus aureus*	
				Fungal pathogens *Candida* spp.	
**Central nervous system**					
Multiplex PCR	Biofire diagnostics	FilmArray BCID	1 h	Gram positive and gram-negative bacteria	
				*Streptococcus species, Haemophilus influenzae, Neisseria meningitidis*	▪ Cannot differentiate between live and dead organisms
				Viruses	
				Cytomegalovirus, enterovirus, herpes simplex viruses 1 and 2, human herpesvirus 6, human parechovirus, and varicella-zoster virus	

### 1.2. Overview of clinical need and advantages of metagenomics

Clinical metagenomics using next-generation sequencing (mNGS) has the potential to surpass the limitations of conventional diagnostics and make a seismic shift in the care of patients suffering from various infections (Simner et al., 2018). Unlike other diagnostic methods, mNGS does not require background knowledge of a suspected pathogen (Duan et al., 2021). mNGS can capture millions to billions of nucleic acids sequences at once and detect multiple organisms including novel pathogens that may be present in a clinical specimen (John et al., 2021). The time required for sample preparation, sequencing, and preliminary bioinformatic analysis depends on the nature of sequencing platform being used (Morsli et al., 2021a,b, 2022a,b). For example, newly available long-read sequencing platforms, such as Oxford Nanopore sequencing, provide real time pathogen detection within minutes and additional information regarding genotyping and bacterial profiling in less than 6 h (Morsli et al., 2021a,b, 2022a,b). Also, Oxford Nanopore Technologies is currently the most prevalent and cost-effective mNGS platform in low- and middle-income countries (Yek et al., 2022). Clinical NGS includes two sequencing strategies: targeted amplicon sequencing and untargeted shotgun metagenomic sequencing. The targeted amplicon sequencing targets the universally conserved regions among bacteria (16S or 23S rRNA gene) or fungi and parasites (internal transcribed spacer (ITS), 18S rRNA, 28S rRNA gene) for pathogen detection (Salipante et al., 2013; Wagner et al., 2018). As an example, PCR-amplified 16S rRNA gene sequencing targets and amplifies one or more selected hypervariable regions (V1–V9) of the 16S rRNA gene. However, the choice of a particular hypervariable region targeted in 16S rRNA gene sequencing appears to be one of the biggest factors underlying technical variation in microbiome composition (Hiergeist et al., 2015; Tremblay et al., 2015; Gohl et al., 2016).

Unlike targeted amplicon sequencing, which only targets specific genes or gene regions, shotgun metagenomic sequencing targets the entire genetic content of a clinical sample, thus permitting the detection of all potential pathogens (Chiu and Miller, 2019). The capability to simultaneously identify viruses, bacteria, fungi, and parasites in a sample makes it broadly appealing for co-infection cases (Chen et al., 2021a). Furthermore, the amount of information derived from shotgun mNGS sequencing can potentially be used for additional analyses, such as antibiotic resistance profiling, virulence gene information, metabolic function profiling, and analyses of human host responses via transcriptome profiling (Chiu and Miller, 2019).

### 1.3. Commercially available clinical metagenomics platforms

Recently mNGS testing and analyses have become commercially available. For example, Charles Chiu and colleagues from the University of California, San Francisco (UCSF) are pioneers in the development of mNGS testing for the diagnosis of central nervous system (CNS) infections (Wilson et al., 2014). In 2014, the first use of mNGS was reported for the diagnosis of neuro-leptospirosis on CSF from a 14-year-old boy presenting the signs of meningoencephalitis (Wilson et al., 2014). This was the first report demonstrating the use of mNGS with medically actionable information and successful clinical diagnosis that led to the appropriate treatment of the patient (Wilson et al., 2014). Since then, UCSF provides validated mNGS DNA and RNA testing for patients with meningitis and/or encephalitis. The UCSF diagnostic lab also offers mNGS DNA testing for patients with sepsis and disseminated infections (Chiu and Miller, n.d.). UCSF software analyzes sequence reads, identifies those reads which align to pathogens in the GenBank database, and issues a report showing the presence of pathogens in a clinical sample, along with clinical interpretation. At least 66.7% of pathogens detected from CSF were true positives, and only 5.6% were found to be false positives (Miller et al., 2019). The mNGS test at USCF for pathogen detection from CSF specimens showed a sensitivity of 86.1% and a specificity of 97.9%. The mNGS test for pathogen detection from plasma samples showed a sensitivity of 77% and specificity of 86%. The turnaround time from shipping samples to delivery of a report is generally 1–2 weeks (Chiu and Miller, n.d.).

The Karius test (Karius, California, United States) is another example of how mNGS is useful for the diagnosis of bloodstream infections (BSIs) and sepsis (Blauwkamp et al., 2019). The Karius test involves extraction of cell-free DNA (cfDNA) from plasma, then a sequencing library is created and sequenced using Illumina technology. The sequence data is compared to an internal reference database encompassing a number of microbial genomes (Blauwkamp et al., 2019). A published study by Thair et al. confirmed that the Karius test detected approximately three times more positive cases than culture-based detection (Thair et al., 2017). However, the limitation is that the test can give false positive results. The Arizona-based Fry Laboratories also provides DNA sequencing diagnostic services for cutaneous, gastrointestinal, hematologic, musculoskeletal, and pulmonary infections (Fry, n.d.).

The Beijing Genomic Institute (BGI Genomics), a China-based company, is one of the largest companies that provide clinical mNGS services for the detection of pathogens causing respiratory infections such as Coronavirus and other pathogenic microorganisms (Jiang, n.d.). The sequencing services by the Zhejiang, China-based IngeniGen XunMinKang Biotechnology company also provide the detection of undiagnosed pathogens in patients with respiratory diseases (Wang et al., 2019; Li et al., 2022).

## 2. Detection of pathogens via clinical metagenomics

mNGS is an unbiased culture-independent and hypothesis-free sequencing technology that has shown tremendous clinical application particularly in the diagnosis of CNS infections, bloodstream infections, and respiratory tract infections (Blauwkamp et al., 2019; Miller et al., 2019; Wilson et al., 2019; Chen H. et al., 2020; Hasan et al., 2020; Haston et al., 2020; Li et al., 2020; Chen et al., 2021b; Hogan et al., 2021; Jing et al., 2021; Liu et al., 2021; Mu et al., 2021; Pollock et al., 2021; Zhou et al., 2021; Deng et al., 2022; Fu et al., 2022; Guo et al., 2022; Li et al., 2022; Wang et al., 2022; Zhang et al., 2022; Morsli et al., 2022b). Examples of recent applications of mNGS in the diagnosis of these infections are provided in Table 2. Below is a brief overview of the areas where mNGS has made considerable impact and the implications.

**Table 2:** Examples of the potential impact of clinical shotgun metagenomics (2019–2022) on infectious disease diagnosis.

Study details	Type of the study (*n* = subjects)	Samples and target population	Sequencing platform	Main findings
**Respiratory infections**				
Chen H. et al. (2020)	Prospective, observational study (*n* = 93)	Bronchoalveolar lavage fluid from patients with lower respiratory tract infections	Illumina Nextseq 550	The detection rate of mNGS for causative pathogen of lower respiratory infection was significantly higher (65% vs. 20%) than traditional culture method.
Li et al. (2020)	Prospective study (*n* = 121)	Lung biopsies from patients with peripheral pulmonary lesions and lung infection	BGISEQ-50	The percentage of mNGS-positive samples in radial endobronchial ultrasound (R-EBUS)-guided transbronchial lung biopsy (TBLB) was 78.8% that was significantly greater than TBLB (60.0%).
Zhou et al. (2021)	Multi-center, prospective, observational study (*n* = 159)	Bronchoalveolar lavage fluid from patients with pulmonary infections	Illumina NextSeq 550	mNGS detected more organisms (117 vs. 72) when compared with standard methods including bacteria (89 vs. 54), viruses (10 vs. 3), and fungi (18 vs. 15). Importantly, the bacteria known to cause pneumonia was detected only by mNGS that included *Haemophilus influenzae*, *Legionella pneumoniae*, *Mycobacterium avium*, *Mycobacteroides abscessus*, *Chlamydia psittaci*, and *Actinomyces* species. mNGS also led to the treatment modification for 59 patients.
Azar et al. (2021)	Prospective, observational study, (*n* = 30)	Bronchoalveolar lavage fluid from immunocompromised adults with pneumonia	Illumina NextSeq500 or NextSeq550	A combination of mNGS and conventional testing improved the diagnostic rate of pneumonia from 35% to 58%.
Chen S. et al. (2021)	Single center, retrospective, observation study (*n* = 408)	Blood, sputum, urine and bronchoalveolar lavage fluid from COVID-19 patients	BGISEQ-50	mNGS showed positive detection rate of 92.3% in bronchoalveolar lavage and 66.7% in sputum. Overall, mNGS results were comparable with conventional culture.
Chen Y. et al. (2021)	Retrospective study (*n* = 90)	Bronchoalveolar lavage fluid, transbronchial brushing from patients with focal pulmonary infections	Illumina Nextseq 550	The analysis of patients with focal pulmonary infections revealed sensitivity of mNGS in bronchoalveolar lavage fluid, transbrochial brushing group, and pathological specimen was 50%, 66.7%, and 90%, respectively.
Deng et al. (2022)	Retrospective, observational study (*n* = 103)	Bronchoalveolar lavage fluid from children with pneumonia	Illumina NextSeq CN500 sequencer	Out of 52 monomicrobial and 44 polymicrobial cases, mNGS detected 48 and 29 cases, respectively. Overall, the pathogen detection rate of mNGS was higher than conventional detection methods.
Zhang et al. (2022)	Retrospective, observational study (*n* = 47)	Bronchoalveolar lavage fluid from patients with lower respiratory tract infections	MGISEQ-2000	As compared to conventional culturing, mNGS increased the detection rate for causative pathogens of lower respiratory tract infections with a diagnostic sensitivity of 80% and specificity of 35.13%.
Xu et al. (2022)	Retrospective, observational study (*n* = 35)	Alveolar lavage fluid or venous blood from patients with severe psittacosis pneumonia	DA8600	mNGS detected DNA of *chlamydia psittaci* in alveolar lavage fluid of 30 patients and blood of 5 patients.
Pollock et al. (2021)	Single-center, proof-of-concept study (*n* = 30)	Plasma samples from patients with pulmonary tuberculosis	Illumina NextSeq 550	*Mycobacterium tuberculosis* cell-free DNA was detected from the plasma of 50% of pediatric and 60% of adult patients. Furthermore, it was also detected in an additional 25% of pediatric and 40% of adult patients when the relaxed research use statistical threshold was applied.
Mu et al. (2021)	Single-center, prospective study (*n* = 292)	Bronchoalveolar lavage fluid and sputum from patients with different kinds of lower respiratory tract infections	Nanopore	Compared with conventional testing, mNGS showed 96.6% sensitivity and 80% specificity and detected pathogens in 63 out of 161 culture-negative cases. Furthermore, mNGS proposed antibiotic de-escalation for 34 patients.
Li et al. (2022)	Single-center, prospective study (*n* = 138)	Bronchoalveolar lavage fluid from patients with pulmonary infections	Illumina Miniseq	mNGS improved diagnosis by detecting more pathogens such as bacteria (53 vs. 27) and viruses (16 vs. 1) than conventional methods. Importantly, mNGS led to the treatment modification for 34 out of 138 patients.
Guo et al. (2022)	Single-center, retrospective study (*n* = 121)	Bronchoalveolar lavage fluid from children with community-acquired pneumonia	Illumina Novaseq	The causative pathogens of pneumonia were only detected by mNGS. These organisms included *Streptococcus pneumoniae*, *Mycoplasma pneumoniae*, *Haemophilus influenza*, Human bocavirus 1, and *Mycobacterium tuberculosis*. Moreover, mNGS identified 50% of human bocavirus-infected cases which were co-infected with other bacteria of respiratory origin.
**Bloodstream infections**				
Hogan et al. (2021)	Multicenter, retrospective study (*n* = 82)	Plasma samples from patients with suspicion of several infections	Illumina	The positivity rate for Karius-based mNGS was 61.0%. Of which 50% of cases were detected with monomicrobial infections and 50% of them were infected with 2 or more organisms. Overall, Karius-based mNGS showed a positive impact on 7.3% of cases, a negative impact on 3.7% of cases, and showed no impact on 86.6% of cases.
Blauwkamp et al. (2019)	Prospective study (*n* = 350)	Plasma samples from patients with clinical suspicion of sepsis	Illumina NextSeq 500	In contrast to culture, mNGS identified much more bacteria. 62 out of 166 samples were negative by traditional testing but sequencing identified these microorganisms in cell-free DNA.
Kalantar et al. (2022)	Prospective study (*n* = 221)	Blood and plasma from critically ill patients	Illumina Novaseq 6000	The pathogen detection in plasma by mNGS and traditional testing varied by organism. For example, mNGS showed 100% sensitivity for *Staphylococcus aureus* and *Escherichia coli*. However, mNGS missed the detection of *Streptococcus pyogenes*. Furthermore, the findings suggest that detection of a pathogen alone is not sufficient for sepsis diagnosis, instead when combined with hosts transcriptional profiling it may provide promising diagnostic utility.
Wang et al. (2022)	Retrospective, observational study (*n* = 435)	Blood, tissues, urine, sputum and different types of body fluids from patients with clinical suspicion of infections	Illumina NextSeq CN500 sequencer	The overall sensitivity of mNGS results were significantly higher than traditional methods. However, there was no difference in specificity of two methods. The sensitivity of mNGS for bronchoalveolar lavage fluid was 72.6% that was higher than blood that showed mNGS sensitivity of 39.3%.
Liu et al. (2021)	Prospective study (*n* = 24)	Blood samples from patients with hematological malignancies and sepsis	MGISEQ-200	The pathogen detection rate of mNGS was comparable with conventional testing for 9 out of 24 patients. However, for 10 patients, mNGS identified additional pathogens as compared to traditional methods most of the identified pathogens were viruses.
Jing et al. (2021)	Retrospective study (*n* = 209)	Blood samples from patients with suspected bloodstream infections	Illumina NextSeq 550	mNGS of plasma improved the clinical sensitivity (87.1%) and specificity (80.2%) as compared to conventional testing.
Fu et al. (2022)	Single center, retrospective study (*n* = 175)	Blood samples from patients with fever of unknown origin	BGISEQ-2000	mNGS increased the detection rate of new organisms in patients with fever of unknown origin by 22.9 and 19.79% than culture and standard detection methods, respectively. Specifically, it improved the detection rate of bloodstream infections by 38 and 32% respectively, as compared to culture and conventional testing.
**Central nervous system infections**				
Wilson et al. (2019)	Multicenter, prospective study (*n* = 204)	Severely ill pediatric and adult patients admitted to the intensive care unit	Illumina Hiseq	mNGS improved diagnosis over traditional methods of neurologic infections by identifying 22% (13 out of 58) of unique pathogens that were missed by clinical testing. The identification of these pathogens led to the treatment modification of 50% (7 out of 13) of these patients.
Miller et al. (2019)	Development and prospective study (*n* = 115)	CSF samples from patients with meningitis and/or encephalitis and patients with suspected neurological infections	Illumina Hiseq and Illumina MiSeq	For 95 samples, mNGS revealed 73% sensitivity and 99% specificity as compared to conventional testing. Moreover, for 20 CSF samples collected from pediatric patients 92% sensitivity and 96% specificity was observed relative to microbiological testing of CSF.
Hasan et al. (2020)	Retrospective study (*n* = 83)	Hospitalized children with suspected CNS infections	Illumina Miseq	In contrast to conventional methods, mNGS showed 100% diagnostic accuracy, 95% sensitivity, and 96% specificity for cerebrospinal fluid samples for hospitalized patients.
Morsli et al. (2022b)	Prospective and proof-of-concept study (*n* = 52)	Patients with community-acquired meningitis	MinION	Out of 52 subjects enrolled, 47 patients showed positive results on CSF samples via routine diagnostics and MinION sequencer. However, in addition to pathogen detection MinION sequencer provided additional information about genotype and antibiotic susceptibility of pathogens.
Haston et al. (2020)	Prospective study (*n* = 20)	Children with encephalitis of unidentified etiology	Illumina Miseq or NextSeq 500	mNGS detected sequence reads of pathogens such as *Mycoplasma bovis*, *Neisseria meningitidis*, *parvovirus B19,* and *Balamuthia mandrillaris* in 6 out of 20 patients. Furthermore, mNGS also detected some nonpathogenic organisms such as *Cladophialophora* species, human bocavirus, and tobacco mosaic virus. The patients with detectable pathogens via mNGS presented immune-mediated phenomena than patients for whom mNGS did not make any diagnosis.
Chen et al. (2021b)	Retrospective study (*n* = 88)	Patients suspected of encephalitis and meningitis	BGISEQ-50 and MGISEQ-2000	mNGS of cerebrospinal fluid detected pathogens in 56.81% (50 out of 88) of patients. The outcomes of mNGS helped in the treatment modification for 23.9% of patients and provided confidence in the continuation of original treatment for 34.1% of patients.

We used predefined filters to refine PubMed search on “classical article,” “clinical study,” “observational study,” “randomized controlled trial,” and “validation study” from 2019 to 2023. We included only retrospective or prospective clinical studies focusing on hospitalized patients using shotgun metagenomics. We have used the keys words “metagenomics AND respiratory infections” for respiratory diseases. Similarly, “metagenomics AND blood infections” and “metagenomics AND central nervous system infections” for blood stream infections and central nervous system infections, respectively. We also search these same terms in google scholar search and added clinical studies and studies focusing on hospitalized patients using shotgun metagenomics not found in PubMed.

### 2.1. Respiratory infections

Pneumonia is considered among the top 10 causes of death in the United States, especially among immunocompromised patients such as those with hematologic malignancy or undergoing hematopoietic stem cell transplant (Lippert et al., 2022). The identification of the causative agent of pneumonia is difficult and often inaccurate due to the pathogen diversity, heterogeneity of sampling, and limited detection methods (Buchan et al., 2022). Traditional molecular diagnosis for pneumonia is pathogen-specific but unreliable for novel or unexpected pathogens (Diao et al., 2022). The ability of mNGS to provide a comprehensive view of pathogens makes it useful in the diagnosis of unexplained pneumonia and disease of unknown etiology (Ramesh et al., 2019; Diao et al., 2022). Recently, mNGS has improved the diagnosis of pulmonary infections over traditional methods by detecting a broad range of organisms including bacteria, viruses and fungi in a number of recent investigations (Chen H. et al., 2020; Li et al., 2020; Mu et al., 2021; Deng et al., 2022; Li et al., 2022; Zhang et al., 2022). Remarkably, the causative agent was identified only by mNGS in two recent studies (Zhou et al., 2021; Guo et al., 2022). Importantly, mNGS led to the treatment modifications and guided treatment decisions for 127 patients with pulmonary infections (Mu et al., 2021; Zhou et al., 2021; Li et al., 2022). Moreover, mNGS detected 50% of cases coinfected with bacteria of different respiratory origin in another study (Guo et al., 2022).

A mNGS approach can be superior to traditional methods for pathogen detection and confirmation of respiratory infections, particularly for *Mycobacterium tuberculosis* (Jin et al., 2022). *Mycobacterium tuberculosis*, can be quite challenging to detect, however, it has been shown in the last few years that mNGS could potentially be used as the first-line diagnostic test for tuberculosis. Karius-based mNGS testing of plasma samples detected suspected tuberculosis in 60% of adults and 50% of pediatric patients (Pollock et al., 2021).

Lastly, RNA viruses are also considered one of the primary causes of respiratory infections (Miller and Chiu, 2022). mNGS can detect a number of viruses that are usually not screened for in respiratory infections using routine diagnostic assays (Prachayangprecha et al., 2014; Bohl et al., 2022). It has shown good sensitivity and specificity compared to conventional testing and can identify viruses such as Influenza, Rhinovirus, and HIV (Jia et al., 2021). An additional advantage to mNGS is the potential to document and describe emerging, and re-emerging viral infections associated with outbreaks (Quer et al., 2022). For example, RNA-based viral metagenomics has detected the presence of novel human coronavirus variants from patients with respiratory symptoms (Wu et al., 2020; Castañeda-Mogollón et al., 2021).

### 2.2. Bloodstream infections

In 2017, it was estimated that 48.9 million cases and 11 million deaths were related to sepsis globally (Rudd et al., 2020). Thus, the early and accurate diagnosis of BSI is critical to initiate appropriate antibiotic therapy and for patient survival. Recent findings indicate sequencing microbial cfDNA using mNGS is a valuable approach for the detection of BSI pathogens when the conventional diagnostics fail to detect the etiological agent (Hogan et al., 2021; Eichenberger et al., 2022). A retrospective multi-center study utilizing the cfDNA and RNA showed that mNGS had a positive impact in 7.3% of cases, a negative impact in 3.7% of cases, and no impact in 86.6% of cases in patients with suspicion of multiple infections (Hogan et al., 2021). Another study applied mNGS on cfDNA in septic and non-septic intensive care unit (ICU) patients and was able to diagnose sepsis and predicted mortality as soon as the first day (Jing et al., 2022). Similarly, cfDNA of relevant pathogens was detected in the blood plasma of cystic fibrosis patients (Barrett et al., 2020). mNGS testing improved the detection rate of BSI in patients having fever of unknown origin or patients with suspected BSI from 38% to 87.1% when compared to conventional methods (Jing et al., 2021; Fu et al., 2022; Wang et al., 2022). However, no difference was observed in specificity between two methods for patients with clinical suspicion of infections (Wang et al., 2022). In one report, mNGS pathogen detection rate was comparable with routine diagnostics in 37% of cases (Liu et al., 2021). In some scenarios, the pathogen detection rate for mNGS varied by organism. For instance, mNGS was 100% sensitive for the detection of *Staphylococcus aureus* and *Escherichia coli.* However, mNGS test missed the presence of *Streptococcus pyogenes* (Kalantar et al., 2022). In contrast, 37% of BSI cases were found to be positive by only mNGS test in patients with clinical suspicion of sepsis in another study (Blauwkamp et al., 2019).

### 2.3. Central nervous system infections

Neuroinflammatory diseases such as meningitis and encephalitis can be diagnostically challenging due to the requirement of invasive procedures for CSF collection, limited availability/low volume of CNS samples, and difficulty of detection by traditional culture (Vetter et al., 2020; Heming et al., 2022; Mokhtari et al., 2022). Furthermore, meningoencephalitis is related with increased risk of morbidity and mortality and thus needs prompt diagnosis and disease management (Ramachandran and Wilson, 2020). CSF culture is considered the gold-standard method for the diagnosis of meningitis. However, prior antibiotic therapy may reduce the sensitivity of CSF cultures, increasing the possibility of false-negatives (Greenberg and Herrera, 2019). mNGS has potential to detect pathogens of unknown etiology as evidenced by clinical series demonstrating the success of mNGS in the detection of hard-to-diagnose CNS infection cases (Miller et al., 2019; Wilson et al., 2019; Hasan et al., 2020; Haston et al., 2020; Chen et al., 2021b; Morsli et al., 2022b). Recently published studies have confirmed the diagnostic sensitivity of mNGS ranging 22% to 95% in patients with CNS infections (Miller et al., 2019; Wilson et al., 2019; Hasan et al., 2020). mNGS testing guided treatment decisions and clinical actionable management for 34.1%–53% of patients in these reports (Wilson et al., 2019; Chen et al., 2021b). However, contaminants from skin flora can lead to false positive bacterial sequences in CSF specimens obtained by lumbar puncture.

## 3. Limitations, knowledge gaps, and potential solutions of clinical metagenomics

As demonstrated in the previous sections, mNGS is a promising diagnostic technology. However, in the present state of knowledge, metagenomics is not always well-positioned to assist clinicians in rapid clinical decision-making due to expertise required for sample preparation, sequencing, bioinformatic analysis, and the high variability in methodologies and interpretation.

Pathogen detection by mNGS depends on the proportion of pathogen sequences in the total sequencing library. Essentially, the diagnostic performance of mNGS is optimal when the sequencing library contains a nominal fraction of host DNA or there is an enrichment of pathogenic sequences (Olausson et al., 2022). While mNGS tends to be more sensitive than traditional methods as evidenced by several studies (Duan et al., 2021; Guo et al., 2022; Li et al., 2022; Zhu et al., 2022; Wang et al., 2023), one of the caveats to being more sensitive is that mNGS can pick up microbial contamination derived from the environment, containers, reagents, and colonizing microorganisms in the human body, thus giving false-positive results. The use of negative controls is recommended for reagents and containers (López-Labrador et al., 2021). Moreover, depending on the sample source and bacterial load, in most cases the majority of reads in a mNGS data set can be derived from human DNA, while the proportion of pathogens tends to be very low (Gu et al., 2019). In order to overcome this challenge, depletion of host background DNA or targeted sequencing approaches such as depletion of abundant sequences by hybridization (DASH), and finding low-abundance sequences by hybridization (FLASH) in a combination of mNGS could be used (Gu et al., 2016; Hasan et al., 2016; Gu et al., 2019; Quan et al., 2019). For example, a positive selection probe-based system called virome capture sequencing platform for vertebrate viruses (VirCapSeq-VERT) has been established by a research group from the United States to increase the sensitivity of detecting viral sequences in clinical samples (Briese et al., 2015). Compact aggregation of targets for comprehensive hybridization (CATCH) is another available method to capture diverse targets from diverse patient samples (Viral Hemorrhagic Fever Consortium et al., 2019). The sequencing depth required to detect and characterize the genome of interest is a major influencer of metagenomics sensitivity. However, there is no consensus for how sequencing depth should be reported. For now, the choice of sequencing depth is dependent on budget and desired outcomes (Greninger, 2018). Retrospective experiments testing different human specimens with known infection at various sequencing depths are required to determine the ideal sequencing coverage for the diagnosis of different human specimens and infectious agents.

On that note, NGS tests do not equally detect all pathogens. For example, the detection rate of intracellular pathogens in clinical samples, such as a *Mycobacterium,* is relatively low as the amount of cell-free DNA of intracellular pathogen released into extracellular body fluids is relatively minute (Chen P. et al., 2020). The use of higher sequencing depth may help in identifying the presence of less abundant pathogens in a clinical sample. However, as a result of higher sequencing depth, a large amount of data would be generated, and thus, require more time for analysis. To avoid this challenge, rapid and more advanced bioinformatic tools must be developed (Chiu and Miller, 2019; Miller et al., 2020).

The human is a host to several commensal organisms, thus separating organisms associated with true bloodstream infection from transient gastrointestinal or oral flora in blood/plasma samples is an obstacle to the interpretation of mNGS results (Chiu and Miller, 2019; Chen et al., 2022). Retrospective studies using different threshold levels (such as a cut-off value for the abundance of pathogens, number of sequencing reads to detect a specific pathogen, and sequencing read normalization) are required to differentiate potential pathogens from commensal organisms. Moreover, the clinical significance of identified organisms needs to be further confirmed by conventional testing and the condition of the host (Chen et al., 2022). If a patient presents with an infection and receives appropriate curative therapy, mNGS can still detect lingering DNA from dead pathogens. It is unknown how long the detectable half-life of a pathogen is once the patient receives appropriate treatment and is conceivably circumstantial. A potential solution is the detection of RNA, whose abundance is directly correlated with the degree of gene transcription activity, thus, it can distinguish dead and live organisms in a clinical sample (d’Humières et al., 2021). Compared with DNA sequencing, a combination of DNA and RNA sequencing may have additional benefits (Arroyo Mühr et al., 2021). However, the detection of RNA through mNGS still has its challenges because of the higher abundance of human-derived RNA in a clinical sample as well as the labile nature of RNA. To overcome this challenge, depletion of the host background is needed (Zheng et al., 2021).

Another limitation of the technology is the actual determination of what is detected (López-Labrador et al., 2021). For example, incomplete databases, mis-annotated sequences, databases containing contaminating organisms, bias in databases, and misclassification of organisms all affect the actual determination of the pathogen after sequencing (López-Labrador et al., 2021; Diao et al., 2022). Moreover, differences in pipelines, reproducibility, quality control, and workflow may lead to different and inaccurate pathogen identification between hospitals and commercially available mNGS tools (López-Labrador et al., 2021). Other logistical challenges include patient privacy, bioinformatic data storage, and lack of standardization (Diao et al., 2022). Continuous effort is needed to improve academically and commercially available tools and make them more accessible to the public (Chen et al., 2022). For example, efforts for improving crowdsourcing of bioinformatics pipelines, software, and taxonomic metagenome profilers could be made. Furthermore, stringent quality controls in the laboratory such as unidirectional workflow, strict decontamination methods during nucleic acid processing, and use of negative controls can help in reducing the detection of exogenous DNA contamination derived from reagents and laboratory environment (Chen et al., 2022).

Currently, the role of mNGS is mostly derived from case reports and small cohort studies (Zhang et al., 2020). Large-scale clinical and cross-institutional studies are required to validate the clinical efficacy of mNGS and to provide a better understanding of how metagenomic approaches could help us to improve patient outcomes over the current standard of care (Zhang et al., 2023). While mNGS may be more expensive than routine diagnostics, the incremental cost is minimum when compared to the cost of invasive diagnostic procedures, a series of diagnostic tests, and cost of intensive care units in hospital; thus, it may help in reducing the overall health care resources (Miller et al., 2020).

## 4. Conclusions and future perspectives

In conclusion, the diversity in clinical metagenomics methods, although allowing flexibility, translates to variability in application and performance relative to conventional diagnostics. Given sensitivity and specificity of mNGS is influenced by a number of factors including the sample type, quantity of host DNA, the sequencing platform used, number of reads generated, selected reference database, and data analysis tools, as well as issues surrounding the current cost and expertise limitations, it is unlikely clinical metagenomics will be utilized as a first-line approach unless these issues are resolved. Thus, an important question in clinical metagenomics remains: Should mNGS only be used as a last resort when gold-standard culture-based or current molecular diagnostic methods fail? Or are there benefits to using it at earlier stages in certain populations?

At this juncture, clinical settings where copious resources are already expended or where patients are at risk for mortality from rare or difficult to treat pathogens, such as in critically ill or immunosuppressed patients (transplant, cancer etc.), could exemplify practical target populations for implementing clinical metagenomics as a standard of care. Despite unresolved application questions, mNGS has undeniable benefits over traditional testing and potentially provides a more complete picture of the state of any clinical infection. Thus, the combination of mNGS and conventional diagnostic methods could be a superior diagnostic strategy to improve overall public health and healthcare associated costs. Moreover, mNGS may vital for time-sensitive diagnostics in life-threatening infections. With advancements in sequencing technology, clinical metagenomic sequencing can not only identify the pathogen but also predict antimicrobial resistance and virulence, enabling prompt and effective treatment. However, to fully utilize mNGS as a clinical diagnostic tool, it is essential to standardize the methods, bioinformatics, and databases used in practice.

## Author contributions

JG-P conceived and supervised the review topic. All authors contributed to the article and approved the submitted version.

## Funding

This work was partially supported by NIH 1K01AI143881 to JG-P.

## Conflict of interest

The authors declare that the research was conducted in the absence of any commercial or financial relationships that could be construed as a potential conflict of interest.

## Publisher’s note

All claims expressed in this article are solely those of the authors and do not necessarily represent those of their affiliated organizations, or those of the publisher, the editors and the reviewers. Any product that may be evaluated in this article, or claim that may be made by its manufacturer, is not guaranteed or endorsed by the publisher.
